# Association of COVID-19 Real-Time Reverse Transcription-Polymerase Chain Reaction (RT-PCR) Cycle Threshold Value With Surrogate Markers of Disease Severity

**DOI:** 10.7759/cureus.31034

**Published:** 2022-11-02

**Authors:** Jyoti E John, Dnyanesh B Amle, Roshan Takhelmayum, Niranjan Gopal, Meena Mishra, Prashant Joshi, Bharatsing Rathod, Rasika Gadkari

**Affiliations:** 1 Biochemistry, All India Institute of Medical Sciences, Nagpur, Nagpur, IND; 2 Microbiology, All India Institute of Medical Sciences, Nagpur, Nagpur, IND; 3 General Internal Medicine, All India Institute of Medical Sciences, Nagpur, Nagpur, IND; 4 Pathology, All India Institute of Medical Sciences, Nagpur, Nagpur, IND

**Keywords:** prognostic marker, ast, ldh, d-dimer, ferritin, ct value, covid-19

## Abstract

Introduction

The cycle threshold (Ct) value in real-time reverse transcription-polymerase chain reaction (RT-PCR) serves as a criterion to diagnose coronavirus disease 2019 (COVID-19) and is inversely proportional to viral load. Levels of inflammatory markers such as aspartate aminotransferase (AST), ferritin, D-dimer, high sensitivity C-reactive protein (hs-CRP), and lactate dehydrogenase (LDH) are used as quantitative measures of COVID-19 severity. We examined the association between these markers and Ct values.

Methodology

This retrospective data analysis included 400 patients with positive RT-PCR results for COVID-19 who were admitted to a tertiary care hospital. Clinical and biochemical data were accessed from the hospital information management system. Associations of clinical parameters and markers of disease severity (e.g., polymorph, AST, hs-CRP, D-dimer, LDH, and ferritin levels) with Ct values were assessed.

Observations

LDH, ferritin, D-dimer, and hs-CRP were found to be significantly higher in moderate and severe groups than in the mild COVID-19 group. AST, ferritin, and hs-CRP levels were also significantly higher in severe COVID-19 subjects, compared to moderate COVID-19 subjects. Ct values for the E (envelop) gene and ORF (open reading frame) 1b gene were found to be significantly higher in those with severe COVID-19. Polymorph counts in subjects with Ct values of 25 or higher were significantly increased, compared to those with Ct values under 30. LDH, D-dimer, and hs-CRP levels in subjects with Ct values over 30 were significantly lower than for those with Ct values under 30. Ferritin was the best independent predictor of non-survival in study subjects, with an area under the curve (AUC) of 85.5% (95% confidence interval = 73.2-95.9). The Ct value for the E gene had an AUC of 75.1%, and the ORF1b gene had an AUC of 64.5%. However, no significant correlation was detected between any parameter and Ct value.

Conclusion

Polymorph, LDH, ferritin, D-dimer, and hs-CRP levels were significantly elevated in subjects with low E gene Ct values. Also, these subjects were at risk of severe disease and fatality. Ct values for the E gene thus could serve as an early indicator for patients at risk of severe disease and death.

## Introduction

The severe acute respiratory syndrome caused by severe acute respiratory syndrome coronavirus 2 (SARS-CoV-2) represents the greatest pandemic of the 21st century. As of July 2022, India stands second only to the United States with regard to diagnosed cases, with 4,35,00,000 cases. Concerning the death toll, India ranks third worldwide [1]. The most common method to diagnose coronavirus disease 2019 (COVID-19) is real-time reverse transcription-polymerase chain reaction (RT-PCR) in conjunction with clinical factors. The cycle threshold (Ct) count as determined by RT-PCR is inversely proportional to viral load, which in turn can help determine clinical progress and outcome [2].

Inflammatory markers such as the serum cytokines lactate dehydrogenase (LDH), ferritin, D-dimer, interleukin-6, and high sensitivity C-reactive protein (hs-CRP) are used as quantitative measures of the severity of COVID-19. CRP is among the first biomarker to be altered in the inflammation process and thus is a potentially useful predictor of disease outcome in COVID-19 [3]. Elevated liver enzymes indicate hepatic damage; specifically, aspartate aminotransferase (AST) has been proposed to be elevated in subjects with severe COVID-19. The tropism of the virus towards angiotensin-converting enzyme-2 (ACE-2) receptors, which are abundant in the liver and bile duct cells, is supposed to be the pivotal factor in liver damage [4]. COVID-19 also has been associated with coagulation activation, fibrinolysis, and pulmonary microvascular immuno‐thrombosis via the immune-thrombotic pathway. D-dimer, an indicator of this process, has been implicated as a prognostic marker in the disease [5]. As most treatment protocols are for symptomatic COVID-19 patients, it is important to identify biomarkers that can predict complications to improve adverse outcomes among these patients.

Few studies have evaluated the association between the clinical severity of COVID-19 and Ct values. Some studies have reported low Ct values in patients with COVID-19 associated with increased levels of inflammatory biomarkers creatinine kinase and high-sensitivity troponin I and lower levels of lymphocyte, T-cell, and serum albumin [6]. Ct value also has been associated with demographic parameters and infectivity. The E (Envelope) gene and ORF (Open reading frame) 1b gene are used most commonly to diagnose COVID-19. [7]. One study has reported a scoring system in which Ct values are useful predictors of severity and outcomes in COVID-19 patients [8]. However, few studies have refuted the association between Ct value and clinical severity [9]. Therefore, the present study assessed the association between inflammatory markers for adverse outcomes in COVID-19 viz. AST, ferritin, D-dimer, hs-CRP, and Ct values for The E gene and ORF1b gene. We also assessed the association between clinical parameters and Ct values.

## Materials and methods

This hospital-based retrospective study was conducted in the Department of Biochemistry in collaboration with the Departments of Medicine and Microbiology at All India Institute of Medical Sciences, Nagpur, India, after approval from the institutional ethical committee (approval number: IEC/Pharmac/2020/189). Participants included subjects with positive RT-PCR tests who were admitted to the hospital. Only symptomatic subjects were admitted to the hospital and subjects with all grades, viz. mild, moderate, and severe symptoms were included in the study. Individuals with a history of any acute inflammatory or debilitating illness (e.g., tuberculosis, cardiovascular, or cerebrovascular disease), ongoing chronic illness (e.g., rheumatoid arthritis, systemic lupus erythematosus, pelvic inflammatory disease, inflammatory bowel disease, connective tissue disorders), or malignancy were excluded.

Sample collection and analysis for RT-PCR were conducted as per standardized procedure [10]. Clinical, laboratory, and outcome data were taken from the medical records department of the hospital. Laboratory data included clinical biochemistry blood investigations and viral load (Ct value on RT-PCR). A total of 400 subjects satisfying the inclusion and exclusion criteria were selected. The participants’ symptoms were categorized as asymptomatic, mild, moderate, or severe as per standard definitions [11].

Routine biochemical parameters were analyzed on a Cobas® 6000 modular (c501 and e601) biochemistry and immunoassay analyzer using appropriate reagent kits (F. Hoffmann-La Roche AG, Basel, Switzerland). The estimation of serum hs-CRP was based on a particle-enhanced immunoturbidimetric assay with a biological reference interval of <5 mg/L. Estimation of serum ferritin was based on an electrochemiluminescence immunoassay with a biological reference interval of 30-400 μg/L in 20-60-year-old males and 13-150 μg/L in 17-60-year-old females. Quality control assessment was conducted via an internal procedure using Cobas PreciControl (F. Hoffmann-La Roche AG, Basel, Switzerland). Plasma D-dimer was estimated on a STAR Max® hemostasis analyzer (Diagnostica Stago SAS, Asnières-sur-Seine, France) and based on an immune-turbidimetric assay with a biological reference interval of <500 ng/mL. Biological reference intervals followed package inserts of the respective diagnostic kits.

Data collection

Rates of illness severity and death among COVID-19 patients during hospitalization were the main outcome measures of the study. All clinical, laboratory, and outcome data were taken from the hospital management information system services provided by the Centre for Development of Advanced Computing India Ltd. at All India Institute of Medical Sciences, Nagpur. Data included demographics, clinical blood biochemistry, and viral load (Ct) of COVID-19.

Statistical analysis

The sample size was finalized at an α error of 5%, power of 80%, and precision of 0.05. The data were analyzed using appropriate statistical methods in IBM SPSS Statistics for Windows, Version 21.0 (Released 2012; IBM Corp., Armonk, New York, United States). Data were analyzed for a test of normality. Continuous variables were expressed as mean ± SD in the parametric distribution of data and as the median and interquartile ranges (25th and 75th percentiles) in non-parametric distributions. Comparisons within groups were performed using Mann-Whitney U or Kruskal-Wallis tests followed by the posthoc Dunn test. Observed frequencies of categorical variables were compared by Chi-square test. The receiver operator characteristic (ROC) curve was plotted to assess the predictive ability of Ct values and biomarkers for survival. Correlation analysis was performed using Spearman’s rank-order correlation analysis. A p-value of <0.05 was considered significant except for the correlation analysis, for which a p-value of 0.01 (two-tailed) was considered significant.

## Results

Among 400 participants, 274 had mild illness, 78 had moderate illness, and 48 had severe illness based on established criteria [11]. During admission, 379 survived and 21 died. Study groups were well-matched for gender distribution (p=0.257). Table 1 summarizes the general characteristics of the study groups. The study population was divided into three groups mild (n=274), moderate (n=78) and severe (48) based on the severity of the illness as per established criteria. The study subjects were also divided into survivors (n=379) and non-survivors (n=21) based on the outcome. 

**Table 1:** General characteristics of participants by illness severity and survival a p<0.05 vs. mild group, b p<0.05 vs. moderate group SBP: systolic blood pressure; DBP: diastolic blood pressure; SPO2: peripheral oxygen saturation; Hb: haemoglobin; Na: sodium; Ct: cycle threshold

Characteristics	Mild (n=274)	Moderate (n=78)	Severe (n=48)	p-value	Survivor (n=379)	Non-survivor (n=21)	p-value
Age (years)	48 (33-58)	56 (44-65)^a^	61 (51-70)^a^	<0.0001	66.5 (50.75-74)	50 (34-62)	0.001
Gender (female)	94 (34.3%)	23 (29.5%)	11 (22.9%)	0.257	6 (28.6%)	122 (32.3%)	0.468
Symptom							
Fever	139 (50.7%)	44 (56.4%)	28 (58.3%)	0.480	203 (53.6%)	8 (38.1%)	0.124
Dry cough	130 (47.4%)	59 (75.6%)	42 (87.5%)	<0.0001	212 (55.9%)	19 (90.5%)	0.001
Breathlessness	25 (9.1%)	55 (70.5%)	45 (93.8%)	<0.0001	106 (28%)	19 (90.5%)	0.578
Fatigue	51 (18.6%)	49 (62.8%)	38 (79.2%)	<0.0001	123 (32.5%)	15 (71.4%)	0.316
Sore throat	119 (43.4%)	30 (38.5%)	24 (50.0%)	0.444	164 (43.3%)	9 (42.9%)	0.578
Loose stools	15 (5.5%)	16 (20.5%)	10 (20.8%)	<0.0001	36 (9.5%)	5 (23.8%)	0.052
Nausea	29 (10.6%)	6 (7.7%)	6 (12.5%)	0.635	39 (10.350	2 (9.5%)	0.633
Runny nose	50 (18.2%)	22 (28.2%)	3 (6.2%)	0.008	73 (19.3%)	2 (9.5%)	0.210
Body aches	36 (13.1%)	7 (9.0%)	10 (20.8%)	0.162	48 (12.7%)	5 (23.8%)	0.130
Headache	38 (13.9%)	13 (16.7%)	8 (16.7%)	0.764	55 (14.5%)	4 (19.0%)	0.376
Anosmia	45 (16.4%)	1 (1.3%)	1 (2.1%)	<0.0001	46 (12.1%)	1 (4.8%)	0.267
Ageusia	28 (10.2%)	0 (0.0%)	2 (4.2%)	0.007	28 (7.4%)	2 (9.5%)	0.479
Comorbidities							
Hypertension	54 (19.7%)	33 (42.3%)	25 (52.1%)	<0.0001	99 (26.1%)	13 (61.9%)	0.001
Diabetes mellitus	21 (7.7%)	29 (37.2%)	36 (75%)	<0.0001	70 (18.5%)	16 (76.2%)	<0.0001
Ischemic heart disease	10 (3.6%)	10 (12.8%)	13 (27.1%)	<0.0001	27 (7.1%)	6 (28.6%)	0.004
Other	10 (3.6%)	26 (23.3%)	12 (25%)	<0.0001	43 (21.3%)	5 (23.8%)	0.373
Tobacco consumption	36 (13.1%)	23 (29.5%)	8 (16.7%)	0.003	66 (17.4%)	1 (4.8%)	0.105
General examination findings							
Duration (days)	7 (5-9)	12 (10-13.5)^a^	8 (5-14)^a,b^	<0.0001	7 (4.5-10.25)	9 (6-10)	0.219
Temperature (^0^C)	35.6 (35-38.025)	36.2 (35.3-38.1)^a^	36 (35.2-37.975)^a^	0.002	35.2 (35.15-36.225)	36 (35-38.1)	0.579
Pulse rate (/min)	92 (88-100)	96 (89-103)	110 (98-112)^a,b^	<0.0001	101 (89.75-110)	96 (88-102)	0.147
Respiratory rate (/min)	20 (18-20)	25 (24-26.5)^a^	34 (29.75-34.5)^a,b^	<0.0001	33 (27.75-34)	20 (19-22)	<0.0001
SBP (mmHg)	120 (112-130)	130 (118-140)^a^	142 (118-164.5)^a^	<0.0001	155.5 (110-188)	124 (114-130)	0.031
DBP (mmHg)	80 (72-82)	82 (78-88)^a^	81 (72-92)	<0.0001	80 (69.75-92)	80 (74-82)	0.908
SPO2 at admission (%)	98 (97-99)	92 (91-93)^a^	85 (79.5-87)^a^	<0.0001	86 (82.5-89.25)	97 (95-99)	<0.0001
Haematological parameters							
Hb (g %)	13.2 (11.97-14.7)	12.6 (11.3-13.6)^a^	13.4 (11.55-14.27)^a,b^	0.003	13.6 (11.6-13.7)	13 (11.6-14.5)	0.881
Leucocytes (10^3^/cumm)	6.01 (4.89-7.3)	6.90 (5.57-8.81)^a^	10.37 (7.19-14.57)	<0.0001	10.87 (4.77-17.61)	6.47 (5.02-7.890)	0.004
Polymorphs (%)	59 (53.75-66)	71 (68-78)^a^	84 (77.75-91)^a,b^	<0.0001	81 (70.25-91.5)	62 (56-71)	<0.0001
Lymphocytes (%)	31 (27-37)	22 (17.5-26)^a^	12 (7.75-19)^a,b^	<0.0001	14 (4-22.75)	29 (21-36)	<0.0001
Neutrophil to lymphocyte ratio	1.96 (1.43-2.62)	3.25 (2.40-4.44)^a^	6.98 (4.15-11.70)^a,b^	<0.0001	6.04 (3.16-22.87)	2.06 (1.6-3.45)	<0.0001
Biochemistry parameters							
Urea (mg/dl)	19 (15-24)	18 (16-24)	37 (18.75-91)^a,b^	<0.0001	91 (19.3-91)	19 (16-24)	<0.0001
Creatinine (mg/dl)	0.78 (0.63-0.9)	0.8 (0.66-0.9)	1.15 (0.77-1.72)^a,b^	<0.0001	1.72 (0.6575-1.72)	0.8 (0.66-0.91)	0.003
Na (mmol/L)	140 (139.6-141)	141 (138.75-141)^a^	132 (124-140)^a^	<0.0001	124 (124-140)	140 (139-141)	<0.0001
Biochemical marker							
Aspartate amino-transferase (IU/L)	23.25 (16.825-29)	18 (16-27)^a^	45 (20-45)^a,b^	<0.0001	45 (23.75-45)	22 (16.3-29)	<0.0001
Lactate dehydrogenase (IU/L)	170.5 (159-188)	321 (272-351)^a^	388.5 (327.75-422.25)^a^	<0.0001	383.5 (312-422)	180 (165-239)	<0.0001
Ferritin (ng/ml)	105 (56.75-186)	423 (367.5-540.5)^a^	958 (728.25-1150)^a,b^	<0.0001	980 (733-1037.5)	156 (66-342)	<0.0001
D dimer (mcg/ml)	100 (100-160)	672 (520-814)^a^	1255 (885-1770)^a,b^	<0.0001	1255 (610-1680)	100 (100-460)	<0.0001
C-reactive protein (mg/L)	4.6 (2.4525-9.5)	49 (32.8-76.325)^a^	72.7 (58-92.75)^a^	<0.0001	73.8 (48.425-86.5)	8.3 (3-21.5)	<0.0001
Ct value							
E gene	31 (29-32)	28 (26-30)	27.5 (24-30.25)^a,b^	<0.0001	27 (23.5-29)	30 (28-32)	<0.0001
ORF 1b gene	29 (26-31)	27 (25-30)	26 (23.75-30)^a,b^	0.011	25 (23.5-29.5)	29 (26-31)	0.024

The median age of subjects with moderate and severe disease was found to be significantly higher than subjects with mild disease. Study groups were found to be matched for gender distribution (p=0.257). Symptoms of dry cough (p<0.0001), breathlessness (<0.0001), fatigue (p<0.0001), loose motions (p<0.0001), and comorbidities such as hypertension (p<0.0001), diabetes mellitus (p<0.0001), and ischemic heart disease (p<0.0001) were found to be significantly higher in frequency in subjects with moderate or severe disease compared to those with mild disease. However, symptoms such as running nose (p=0.008), anosmia (p<0.0001), and ageusia (p=0.007) were associated with mild disease. AST (p<0.0001), LDH, ferritin, D-dimer, and CRP were found to be significantly different amongst mild, moderate and severe COVID-19 subjects (p<0.0001 for all). On further post hoc analysis, LDH, ferritin, D-dimer, and hs-CRP were found to be significantly higher in moderate and severe groups compared to the mild COVID-19 group. AST, ferritin, and hs-CRP were also found to be up significantly higher in severe COVID-19 subjects compared to moderate COVID-19 subjects. Ct values for E gene as well as that for ORF 1b gene were found to be significantly higher in severe COVID-19 groups (Table 1).

**Figure 1:**
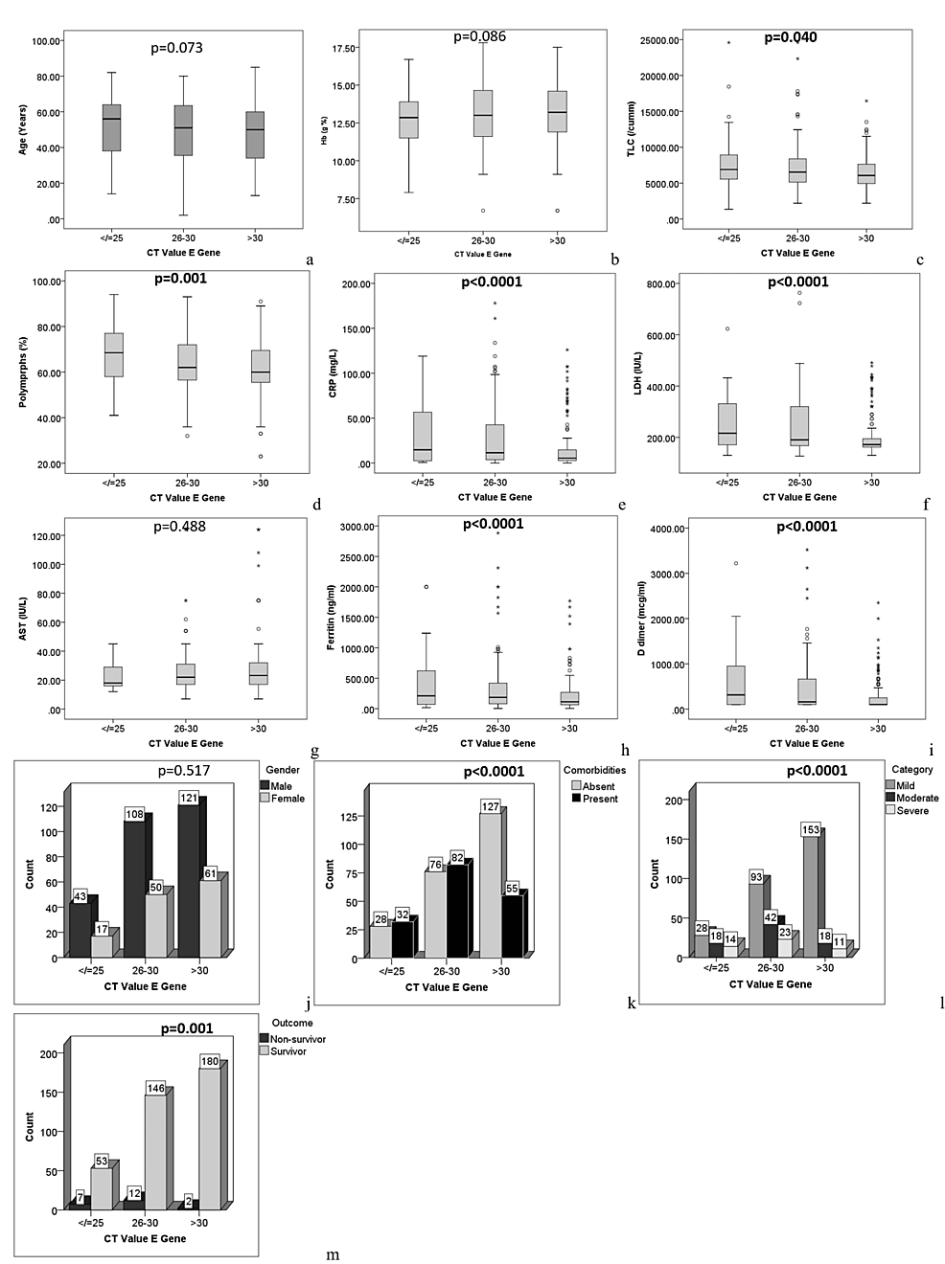
Comparison of demographic, clinical, and laboratory parameters between subjects with different cycle threshold (Ct) values of the E gene Ct: cycle threshold; Hb: haemoglobin; TLC: total leucocyte count; CRP: C-reactive protein; LDH: lactate dehydrogenase; AST: aspartate transaminase

**Figure 2:**
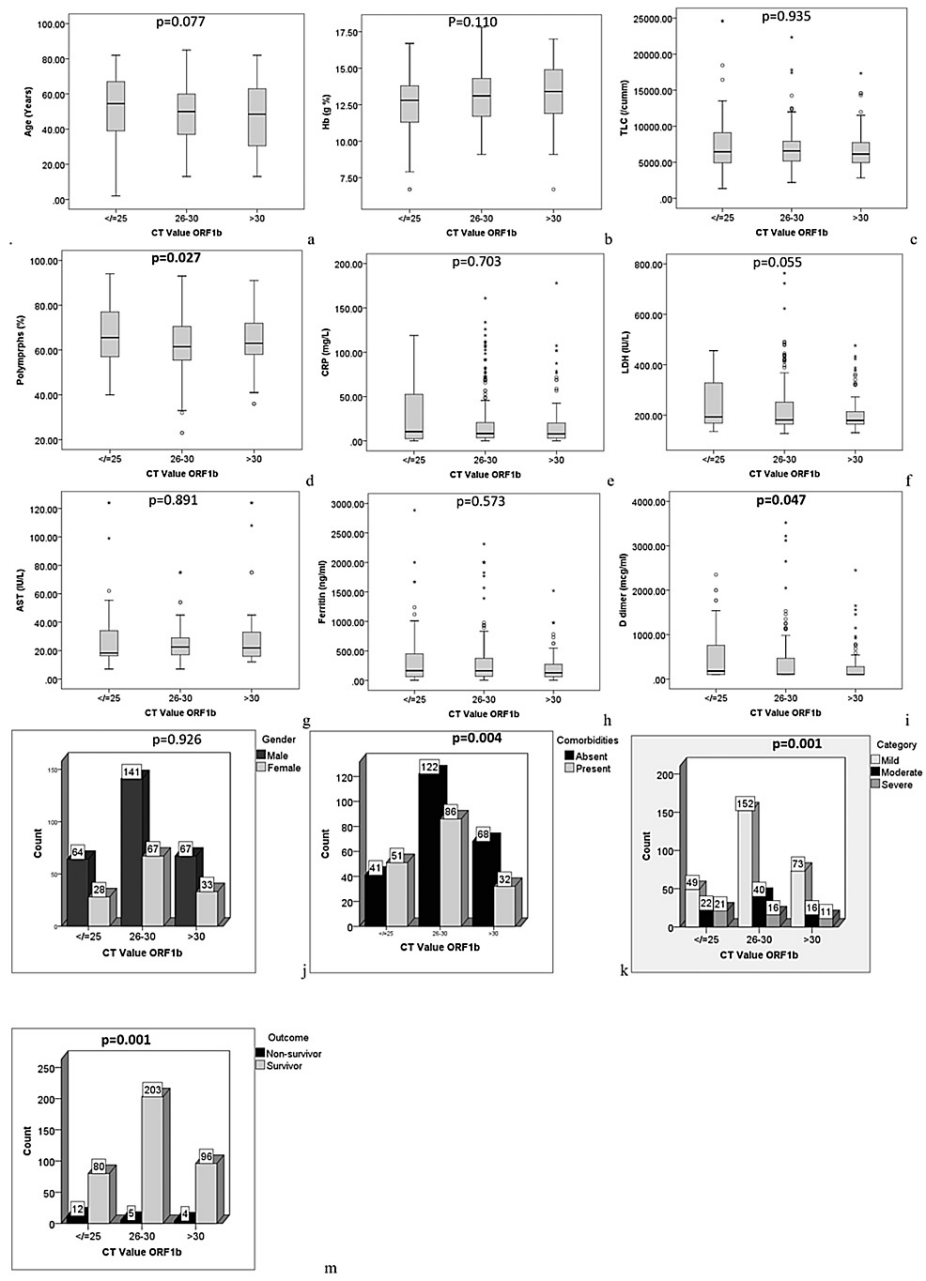
Comparison of demographic, clinical and laboratory parameters between subjects with different Ct value of ORF1b gene. Ct: cycle threshold; Hb: hemoglobin; TLC: total leucocyte count; hs-CRP: high sensitive C-reactive protein; LDH: lactate dehydrogenase; AST: aspartate transaminase

To assess the association between various parameters and Ct value of the E gene and the ORF1b gene, participants were divided into three subgroups based on Ct value (25 or lower, 26-30, and over 30). For each gene group, the Ct subgroups were well-matched for age and gender. For the E gene group, significant differences were noted in levels of total leucocytes (p=0.040), polymorphs (p=0.001), LDH (p<0.0001), ferritin (p<0.0001), D dimer (p<0.0001), and CRP (p<0.0001). The frequency of comorbidities was found to be associated with low Ct value (p<0.0001), severe COVID-19 (p<0.0001), and non-survival (p=0.001). A posthoc analysis showed the polymorph counts in subjects with Ct values of 25 or lower to be significantly higher than in those with Ct values over 30. Also, LDH, D-dimer, and CRP levels in subjects with Ct values over 30 were significantly lower than in those with Ct values under 30. Figure 1 and Figure 2 illustrate the results for each gene, respectively. For the ORF1b gene, significant differences were noted in polymorph (p=0.027) and D-dimer (p=0.047) levels (Figure 2). However, no significant difference was noted in the post hoc analysis (Figure 2).

**Figure 3:**
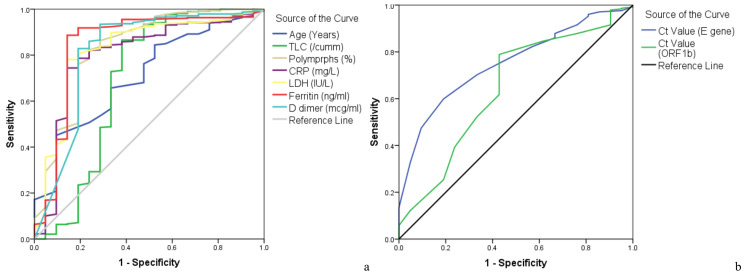
ROC curve analysis of laboratory parameters (a) and Ct value (b) for the predictive ability of non-survival. ROC: receiver operating characteristic; Ct: cycle threshold; TLC: total leucocyte count; CRP: C-reactive protein; LDH: lactate dehydrogenase

Ferritin was found to be the best independent predictor of non-survival in study subjects (AUC=85.5%, 95%C.I.=73.2-95.9). The Ct value for the E gene had an AUC of 75.1% and that of the ORF 1b gene was 64.5%. Table 2 and Figure 3 summarize the results.

**Table 2:** Predictive significance of various parameters in non-survival of COVID-19 subjects Ct: cycle threshold; COVID-19: coronavirus disease 2019

	Area under Curve (%)	Std. Error	p-value	Asymptotic 95% Confidence Interval	
				Lower	Upper
Age (years)	70.8	0.055	0.001	0.600	0.815
Total leucocyte count (/cumm)	68.8	0.084	0.004	0.522	0.853
Polymorphs (%)	83.0	0.055	0.000	0.723	0.938
C-reactive protein (mg/L)	79.9	0.058	0.000	0.685	0.912
Lactate dehydrogenase (IU/L)	82.4	0.053	0.000	0.721	0.927
Ferritin (ng/ml)	84.5	0.058	0.000	0.732	0.959
D dimer (mcg/ml)	81.5	0.064	0.000	0.691	0.940
Ct value (E gene)	75.1	0.046	0.000	0.662	0.841
Ct value (ORF1b)	64.5	0.066	0.025	0.517	0.774

Correlation analysis of various parameters of disease severity with Ct values was performed using Spearman’s rank-order correlation. None of the parameters was found to be correlated with Ct values, regardless of survival or severity subgroup (Table 3).

**Table 3:** Correlation analysis of Ct value of E gene and of ORF1b gene with various biomarkers of disease severity, by survival and by disease severity Correlation expressed in ρ; *p<0.01 (two-tailed significance) Ct: cycle threshold; TLC: total leucocyte count; hs-CRP: high sensitive C-reactive protein; LDH: lactate dehydrogenase

	All (n=400)		Non-survivors (n=21)		Survivors (n=379)		Mild (n=274)		Moderate (n=78)		Severe (n=48)	
	Ct value E	Ct value ORF1b	Ct value E	Ct value ORF1b	Ct value E	Ct value ORF1b	Ct value E	Ct value ORF1b	Ct value E	Ct value ORF1b	Ct value E	Ct value ORF1b
TLC (/cumm)	-0.152*	-0.048	.025	0.048	-0.111*	0.006	-0.054	0.054	-0.006	-0.023	0.078	0.050
Polymorphs (%)	-0.205*	-0.010	-0.021	0.080	-0.180*	0.013	-0.035	0.095	0.041	0.162	-0.037	0.085
hs-CRP (mg/L)	-0.206*	-0.070	0.269	0.376	-0.197*	-0.021	0.000	0.129*	0.150	-0.121	0.047	0.252
LDH (IU/L)	-0.214*	-0.084	0.232	0.086	-0.254*	-0.104*	-0.123*	-0.029	0.097	0.007	0.206	0.225
Ferritin (ng/ml)	-0.180*	-0.105*	-0.025	-0.123	-0.200*	-0.058	-0.051	0.031	0.105	0.011	-0.002	-0.030
D-dimer (mcg/ml)	-0.245*	-0.101*	-0.217	0.139	-0.228*	-0.115*	-0.032	-0.035	-0.061	-0.108	-0.091	0.261

## Discussion

The present study aimed to evaluate clinical and biochemical markers associated with COVID-19 and their relationship with Ct values, which are inversely related to viral RNA load. Disease severity in the study population was categorized as mild, moderate, or severe according to established criteria based on respiratory status and oxygen saturation [11]. We observed an association of increasing severity of illness with a higher frequency of symptoms (e.g., dry cough, breathlessness, fatigue, loose stools) and comorbidities (e.g., hypertension, diabetes mellitus, ischemic heart disease) in subjects with moderate or severe disease, compared to those with mild disease. The results align with those of other studies conducted in central India and China [11,12]. Zhang et al. noted that among 2-11% of subjects with COVID-19 and liver comorbidities, 14-53% had abnormal ALT and AST levels during disease progression [13]. Another study from China reported elevated AST and AST/ALT levels correlated with the severity of COVID-19 and mortality [4]. Our findings also corroborate those from studies showing LDH values to be significantly elevated in severe COVID-19, compared to mild to moderate disease, and in non-survivors, compared to survivors [14,15]. Liver damage may be due to ACE 2 receptors in the liver making it susceptible to SARS-COV-2, systemic inflammation, and hypoxic liver injury, as well as the toxicity caused by pharmacological agents used for treatment [3,4,9,13,16].

Clinical correlation of D-dimer with pro-inflammatory cytokines has been observed in critically ill patients with COVID-19 [16]. D-dimer acts as a surrogate marker for pulmonary damage and thrombo-embolic processes, both of which play important roles in the pathogenesis of COVID-19. Thus, D-dimer may act as a biomarker for severity and prognosis in these patients [17,18]. Warusevitane et al. also have shown strong correlations of CRP levels with disease severity and with lung lesions in patients with COVID-19 [19]. The findings of the current study indicated similar correlations.

The RT-PCR Ct value and the SARS-CoV-2 virus load are inversely proportional to each other, thereby indirectly reflecting the severity of infection [20]. Our results showed that E gene and ORF1b gene Ct values were significantly higher in those with severe COVID-19. A higher frequency of comorbidities was found to be associated with low Ct values for the E gene. This finding is a potential confounder given that comorbidities are associated with severe disease and higher mortality [2]. For the E gene, the polymorph count in subjects with Ct values of 25 or less was found to be significantly higher than in those with Ct values over 30. Also, CRP, LDH, and D-dimer levels in subjects with Ct values of 25 or less were found to be significantly higher than in those with Ct values above 26.

Scola et al. found that a lower Ct value in severe COVID-19 was associated with a high neutrophil count [21], which was corroborated by Yuan et al. [22]. Liu et al. further reported a negative correlation of r=-0.548 between the Ct value and CRP [23]. However, Yuan et al. found no such correlation [22]. Two more recent studies also failed to observe any correlation between the Ct value and D-dimer level [24,25]. Although neither study compared D-dimer values between subjects with different Ct values, the results of both studies support our findings showing significantly higher D-dimer values in subjects with low Ct values. We did not note any significant association of the Ct value for the ORF1b gene with any biochemical parameter.

In our study, Ct values were significantly lower in subjects with severe disease and in non-survivors, compared to their respective counterparts. It has been reported previously that low Ct values are often associated with severe disease and longer viral persistence. A high viral load may increase the severity of the disease and the chances of death [26,27]. In a study of the relationship between Ct values and disease severity or associated morbidity with COVID-19, patients who died had substantially lower Ct values than those who recovered, though the sample size was small [28].

We found ferritin to be the best independent predictor of non-survival in study subjects (AUC=85.5%, 95%CI=73.2-95.9). Ahmad et al. have also reported ferritin as a predictor of mortality (AUC 69%) [29]. Ct value for the E gene had an AUC of 75.1% and that of the ORF1b gene was 64.5%. Few studies have reported Ct value as a predictor with AUC, though many studies assessing the difference in Ct values between survivors and non-survivors have found it to be significant [30].

Despite findings of significant differences regarding various biochemical parameters between subjects with different Ct values, our study failed to find any significant correlation between the studied parameters and Ct values in terms of severity and survival. Due to the retrospective study design and functional problems, we could not evaluate potential confounders such as virus strain or variations in RT-PCR techniques. We also estimated only the relationships associated with the Ct value for the E gene and ORF1b gene and not other genes used in RT-PCR studies around the world for diagnosis of COVID-19. Thus, our study has relevance only in the context of these genes. Nevertheless, our study offers novel insight into the utility of Ct value for establishing disease severity and mortality in COVID-19. These listed points may be deemed as limitations to our study and the findings must be interpreted in light of these factors.

## Conclusions

We conclude that subjects with lower Ct values for the E and ORF1b genes tend to have severe disease and are more susceptible to fatality. Polymorphs and biochemical indicators of disease severity (CRP, LDH, ferritin, and D dimer) were significantly elevated in subjects with lower Ct values for the E gene, but these parameters did not show any correlation with Ct values. Ct values for the ORF1b gene showed no association with any indicator of disease severity. RT-PCR is one of the first investigations to be performed in COVID-19 diagnosis, and the patient’s Ct value is usually known at the time of admission. This information, thus, can serve as evidence to use Ct value as an early and relevant predictor of severity and outcome and can be helpful for the treating clinician. We recommend further investigation of the associations between these parameters and Ct value for other genes used in the diagnosis of COVID-19 using a prospective study design.
